# Osthole Ameliorates Estrogen Deficiency-Induced Cognitive Impairment in Female Mice

**DOI:** 10.3389/fphar.2021.641909

**Published:** 2021-05-06

**Authors:** Frank Adu-Nti, Xu Gao, Jia-Min Wu, Jing Li, Javed Iqbal, Riaz Ahmad, Xin-Ming Ma

**Affiliations:** ^1^ Department of Neuroscience, Key Laboratory of the Ministry of Education for Medicinal Resources and Natural Pharmaceutical Chemistry, National Engineering Laboratory for Resource Development of Endangered Chinese Crude Drugs in Northwest China, College of Life Sciences, Shaanxi Normal University, Xi’an, China; ^2^ Department of Neuroscience, University of Connecticut Health, Farmington, CT, United States

**Keywords:** estradiol, ovariectomy, depression-like behaviors, cognitive deficit, hippocampus

## Abstract

Loss of endogenous estrogen and dysregulation of the estrogen receptor signaling pathways are associated with an increase in risk for cognitive deficit and depression in women after menopause. Estrogen therapy for menopause increases the risk of breast and ovarian cancers, and stroke. Therefore, it is critical to find an alternate treatment for menopausal women. Osthole (OST), a coumarin, has been reported to have neuroprotective effects. This study examined whether OST improves ovariectomy (OVX)-induced cognitive impairment, and alleviates anxiety- and depression-like behaviors induced by OVX in mice. Adult female C57BL/6J mice were ovariectomized and then treated with OST at a dose of 30 mg/kg for 14 days. At the end of the treatment period, behavioral tests were used to evaluate spatial learning and memory, recognition memory, anxiety- and depression-like behaviors. A cohort of the mice were sacrificed after 14 days of OST treatment and their hippocampi were collected for measurement of the proteins of interest using western blot. OVX-induced alteration in the levels of proteins was accompanied by cognitive deficit, anxiety- and depression-like behaviors. OST treatment improved cognitive deficit, alleviated anxiety- and depression-like behaviors induced by OVX, and reversed OVX-induced alterations in the levels of synaptic proteins and ERα, BDNF, TrKB, p-CREB, p-Akt and Rac1 in the hippocampus. Therefore, reversal of OVX-induced decrease in the levels of hippocampal proteins by OST might contribute to the effects of OST on improving cognitive deficit and alleviating anxiety- and depression-like behaviors induced by OVX.

## Introduction 

The permanent cessation of menstruation, called menopause or climacteric, is a natural physiological process for women and causes a series of dysfunctions in the autonomic nervous system due to the loss of hormone fluctuation caused by ovarian failure. Besides the physical symptoms, it is also often associated with a wide array of neuropsychological symptoms, including cognitive deterioration, anxiety, and depression (Hoga et al., 2015). These psychological disorders exert a negative impact on the quality of life of menopausal women in varying degrees (Jenabi et al., 2015). Although estrogen therapy is the mainstay of treatment for the management of menopause symptoms and it is beneficial in ameliorating anxiety and depression (Liu et al., 2019), and improving memory impairment (Phan et al., 2015), a large body of evidence confirms that long-term estrogen therapy increases the risk of breast (Wang et al., 2017) and ovarian cancer, (Beral et al., 2015), stroke (Anderson et al., 2004) and cardiovascular disease (Kim et al., 2020). Therefore, a search for alternative therapies, particularly from herbal medicine is highly desired (Kwee et al., 2007).

Osthole (C_15_H_16_O_3_, 7-methoxy-8-isopentenoxycoumarin, **OST**), a natural coumarin derivative isolated from *Cnidium monnieri (L.) Cusson*, exhibits a series of pharmacological and biological activities, such as anti-inflammatory, antioxidant, anti-apoptotic, anti-tumor, inhibiting microglia proliferation, enhancing neurogenesis, and neuroprotective properties (Nakamura et al., 2009; Hu et al., 2013; Gao et al., 2014; Liu et al., 2015; Xia et al., 2015; Li et al., 2020). OST treatment enhances cell viability, prevents cell death, and increases the reduced levels of synaptic proteins including synapsin-1, synaptophysin (SYP), and PSD95 in cortical neurons induced by Alzheimer precursor polypeptide (APP)-overexpression (Li et al., 2016). OST improves impaired spatial learning and memory in a mouse model of Alzheimer’s disease (Yao et al., 2019). These studies suggest that OST is a promising candidate for reversing estrogen deficient-induced alterations in behaviors.

The hippocampus is the core for memory formation and consolidation in the mammalian brain and is the main target of estrogens (especially 17β-estradiol, E2) (Woolley and McEwen, 1992). The expression of hippocampal synaptic proteins is regulated by E2 treatment in rodents (Li et al., 2004). Notably, hippocampus-dependent spatial reference memory fluctuates with E2 fluctuation during the estrous cycle (Frick and Berger-Sweeney, 2001). OVX impairs spatial learning and memory and induces depression-like behaviors in mice (Li et al., 2004; Lu et al., 2014). Similar to E2, OST has the potential to improve cognitive deficit, and alleviate anxiety- and depression-like behaviors induced by OVX in female mice.

BDNF plays a critical role in spine formation, synaptic plasticity and cognition via binding to its receptor TrkB that couples to an array of signal transduction pathways (Cunha et al., 2010). The serine-threonine kinase Akt and downstream transcription factors such as CREB play a pivotal role in neuronal survival, protection and spine formation, and their activation protects against cellular stress and injury (Tanaka et al., 2000). Akt activation promotes cellular survival, enhancing cognition and spine formation by phosphorylation of CREB, resulting in the up-regulation of CREB target genes (Walton and Dragunow, 2000). CREB plays a critical role in adaptive neuronal responses, in addition to the complex functions in the regulation of learning and memory. BDNF mediates dendrite development and spine formation via fine-tuning of the actin cytoskeletal reassembly (Ravindran et al., 2019). Actin filaments, the major cytoskeletal protein structures in the dendritic spine, play a key role in synaptic plasticity by regulating synapse structure and dendritic spine levels (Cingolani and Goda, 2008). Estrogen regulates spine synapse formation by regulating the actin cytoskeletal network (Spencer et al., 2008). LIM kinase (LIMK) regulates actin polymerization and depolymerization (Bernard, 2007), and plays a key role in cognition (Frangiskakis et al., 1996). LIMK functions as a serine/threonine kinase, and one of its target substrate is cofilin (Yang et al., 1998). Cofilin, a member of the actin depolymerization factor (ADF) family, is a key actin binding protein that promotes the turnover and severing of actin filaments (Maciver and Hussey, 2002), and an increase in the levels of cofilin causes a cognitive deficit in rodents (Deng et al., 2016). Phosphorylation of cofilin by LIMK is important in promoting actin filament extension and dendritic spine formation (Meng et al., 2003), which leads to amelioration of depression-like behaviors and improved cognition. Kalirin-7 (Kal-7) is localized to the postsynaptic side of excitatory synapses, where it interacts with PSD95, GluA1 and TrkB and plays an essential role in the formation of excitatory synapses which is important for maintaining normal cognition (Mandela and Ma, 2012; Yan et al., 2016). Endogenous E2 fluctuations (Spencer-Segal et al., 2011) and/or exogenous administration of E2 (Spencer-Segal et al., 2012) both modulate hippocampal levels of postsynaptic density 95 (PSD-95), phosphorylated AKT (p-AKT) and LIMK1 (p-LIMK1), estrogen receptor (ER)α, BDNF, TrkB (Tuscher et al., 2016) and Kal-7 (Erli et al., 2020). We hypothesized that OST administration reverses both OVX-induced alterations in these proteins and the associated cognitive deficit. The aim of the study was to evaluate whether OST improves OVX-induced cognitive impairment, ameliorates depression-like behaviors and reverses OVX-mediated alterations in synaptic proteins.

## Method

### Animals

9 weeks old female C57BL/6J mice weighing 17–20 g were purchased from Xi’an Jiaotong University Medical School. All animals were housed under standard laboratory conditions with 12 h dark/12 h light cycles, and experiments began 1 week after arrival. They were fed regular mouse chow and given tap water *ad libitum*. Ethical approval was obtained from the College of Life Sciences of Shaanxi Normal University and guidelines for the proper handling of animals were carefully followed including ensuring minimal animal usage and pain.

### Drug

Osthole (purity > 95%) was purchased from the Xi’an Tianyi Biotechnology Co. Ltd. (Xi’an, China).

### Ovariectomy Surgery

Bilateral ovariectomy was performed as described previously (Iqbal et al., 2019). Mice were anesthetized by an intraperitoneal (i.p) injection with pentobarbital sodium. The dorsal part of the lumbar region was shaved and then cleaned with ethanol. One small incision (1 cm) was made through the skin and the muscle wall on each side of the backbone, in the dorsal aspect. Ovaries were then located and removed. The wound was closed in two layers, i.e. muscle and skin using sterile sutures. Sham animals were also anesthetized, the skin and muscle layers were opened and the uterus and ovaries were manipulated but not excised. After surgery, mice were housed individually for 2 h to allow recovery and then re-grouped in their home cages. Successful OVX was evaluated by uterus weight at the end of the experimentation.

### Drug Treatment

**FIGURE 1:**
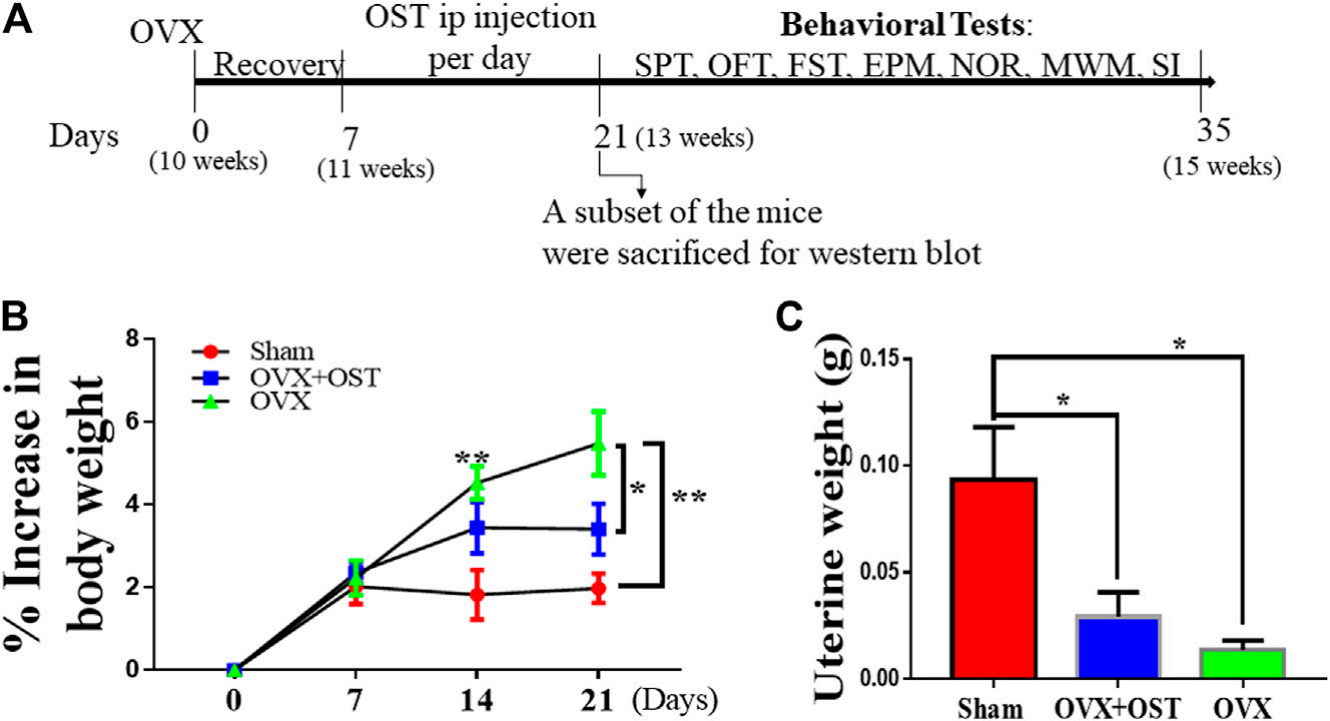
Experimental design **(A)** OVX–ovariectomy; i. p–intraperitoneal; SPT–sucrose preference test; OFT–open field test; FST–forced swim test; EPM–elevated plus maze; NOR–novel object recognition; MWM-Morris Water Maze; SI–social interaction. The age of the mice is shown in brackets. **(B)** The effect of OST on body weight in Sham, OVX, and OVX + OST mice. Body weight gain is shown as percentage of baseline control at day 0 for the different groups at each week. OVX induced a significant increase in body weight gain compared to the sham group, and OST treatment was able to reverse this increase induced by OVX. **(C)** OVX induced a significant decrease in uterine weight compared to the sham. Treatment with OST was unable to reverse this decrease. Data are represented as mean ± SEM (body weight *n* = 20, uterine weight, *n* = 5). *p < 0.05, **p < 0.01 vs sham.

After allowing recovery for 1 week, mice were randomly separated into three groups: i) Sham ii) OVX + saline iii) OVX with osthole (OVX + OST). OST was reconstituted in normal saline and administered via the intraperitoneal route at a dose of 30 mg/kg according to the previous report (Liu et al., 2015) over a period of 14 days. The experimental design is presented in Figure 1A.

### Body Weight

Each mouse was weighed before the OVX surgery (day 0). The weight was taken again on days 7, 14 and 21 post-OVX surgery.

### Behavior Experiments

All the assays to monitor behavior were commenced 24 h after the last OST injection. The mice were kept in the room next to the behavior room in which the experiments were performed for a minimum of 30 min before each test.

#### Sucrose Preference Test

This test was performed as previously described (Yang et al., 2020) with slight modifications. Mice were provided with two bottles containing water and 1% sucrose for 72 h for acclimation. For the SPT, the mice were water deprived for 12 h (07:00 – 19:00). The animals were then given the two bottles containing water and 1% sucrose overnight (19:00 – 07:00). The bottles were weighed at the start and at the end of the test and the sucrose consumption was calculated. The sucrose preference score was calculated as the percentage of sucrose solution ingested relative to the total amount of liquid consumed as determined before and after each test, i.e., sucrose preference = sucrose intake/total liquid (sucrose + water) intake × 100.

#### Open Field Test

The open field box was divided into 16 equal square sections, with 4 central areas and 12 peripheral areas as previously described (Qiao et al., 2017). Mice were placed individually into the center of the open field and left to explore for 5 min during which the activity and behavior were recorded with a digital video camera and Smart v0.06 (Panlab Harvard Apparatus). At the end of each experiment, the OFT box was cleaned with 75% alcohol solution to avoid carryover of olfactory cues. Anxiety-related behavior was deduced from the total distance traveled in the open field, the time spent in the central area, the number of rearings as well as the number of groomings.

#### Elevated Plus Maze

Animals were placed in the EPM under bright light conditions for 5 min as previously described (Zhou et al., 2019). Each arm of the maze measured 12 × 50 cm. The black Plexiglas cross-shaped maze consisted of two open arms with no walls and two closed arms (40 cm high walls) and was on a pedestal 1 m above floor level. Behavior was tracked using an automated system (Smart v0.06, Panlab Harvard Apparatus). Time in the open and closed arms, and the number of arm entries were analyzed.

#### Novel Object Recognition Test

The novel object recognition Test (NORT) was performed as described previously with slight modifications in order to evaluate the short-term memory of the subjects (Ma et al., 2008; Walf et al., 2008). The test was performed in a single day. In the habituation phase, each mouse was placed in the center of an open field box for free exploration for 5 min. In the training phase, two identical objects were placed in adjacent corners of the chamber. Each mouse was granted 5 min to explore the objects in the box. The mice were taken back to their cages after the training to wait for 3 h. In the testing stage, one of the identical objects was replaced with a different but similar object. The mice were allowed to freely explore the two objects for 5 min. The time spent exploring each object was video recorded during the testing phase; exploration of the object was defined as when the mouse oriented toward the object with the distance between the nose and the object less than 1 cm.

#### Morris Water Maze Test

Morris water maze test was performed as described (Zhou et al., 2018). The MWM is widely used for the assessment of spatial learning and memory in animal models. It usually involves two experiments: i) the object location and navigation experiment, where latency is used to measure the learning performance of the mouse; and ii) the spatial probe experiment, where the number of times the mouse passes the original location of the platform is used to measure the memory retention capability of the mouse. In this test, the rodents try to find the platform hidden beneath the cloudy water using visual cues which are located in the surrounding space. The water temperature was set at 25 ± 1°C. The pool was divided into four quadrants. A platform measuring 12 cm in diameter and 20 cm in height was placed 33 cm from the wall of the pool and 2 cm below the water surface in the fourth quadrant. Various visual signs were placed around the water maze pool, and a camera was mounted above the center of the pool to record the motions of the mouse, which were then transferred to a computer. The motions of the mice were analyzed by software (Watermaze version 3.20, ActiMetrics Software) and the latency to reach the platform was recorded. There were 6 days in total for the MWM test. Mice were given one training block daily in the water maze for 6 days. Each training block consisted of four training trials. Animals were given a 60 min inter-trial interval. In each experiment, the mice were released into the pool from one of these points. During the experiment, the animal was allowed to swim freely, find the platform, and remain on the platform for 20 s. The animal was then taken out of the water. The time spent in finding the platform (latency) represented the learning capability and was recorded by camera. The animals were then dried and returned to the cage. On the day of the probe test (day 6), the platform was removed from the pool and each mouse was allowed to swim in the pool for 60 s from the four different starting points. The time spent in the target area and the number of platform crossing were used to evaluate spatial memory.

#### Social Interaction Test

The protocol for this test was adapted from previously published study (Lo et al., 2016). The social approach apparatus was an open-topped box made of acrylic and divided into three chambers with two clear acrylic walls. Dividing walls had retractable doorways allowing access into each chamber. A wire cup was used to contain the stranger mice. Test mice were placed in the central chamber at the beginning of each phase. To initiate each 10 min phase, the doorways to the side chambers were opened, and the mice were allowed to explore freely. During the sociability phase of the test, an unfamiliar mouse (novel mouse 1) was placed in one of the wire cups in a side chamber. During the social novelty phase, a new unfamiliar mouse (novel mouse 2) was put in the wire cup that had been empty during the sociability phase. Exploration of an enclosed mouse or a wire cup was scored when a test mouse oriented toward the cup with the distance between the nose and the cup less than 1 cm, or as climbing on the cup. The time spent in each chamber and time spent exploring enclosed novel mice or empty cups (novel objects) were recorded by a camera mounted overhead, and analyzed by an automated tracking program Supermaze software (Animal Behavior Recognition Technology Development, Shanghai, China).

#### Forced Swim Test

The FST was conducted according to previously published protocols (LaPlant et al., 2009). Animals were placed in the test room for an hour before behavioral testing. Mice were tested in a 4 L Pyrex glass beaker, containing 2 L of water at 25 + 1°C for 6 min. Behavior was video-taped and the last 5 min of the swimming was hand scored by an observer blind to experimental conditions.

### Western Blot

At the end of 14 days of OST administration, a subset of the mice were sacrificed by decapitation and the hippocampi were collected for Western blot as described (Ma et al., 2008). Briefly, after protein extraction, protein samples (25 μg/lane) were separated using 10 and 12% Tris-glycine sodium dodecyl sulfate-polyacrylamide gel electrophoresis and transferred to polyvinylidene fluoride (PVDF) membranes (Millipore, Germany). Each gel contained molecular weight standards plus protein samples from sham, OVX and OVX + OST groups. The membrane was blocked with 5% non-fat milk for 90 min and incubated overnight at 4°C with diluted primary antibodies. Table 1 shows the list of antibodies used in this study. After 24 h, membranes were rinsed three times, incubated with secondary antibodies for 60 min at room temperature, and visualized using the Luminescent Imaging (Tanon 6200 Luminescent Imaging Workstation, Tanon, China). Protein bands were quantitatively analyzed, and all signals were normalized within the same membrane to GAPDH.

**TABLE 1:** List of antibodies used in the study.

Name of antibody	Catalog number	Company
Kalirin 7	JH2958	University of Connecticut health center
PSD95	AF7839	Affinity
GluA1	13,185	Cell signaling
GAPDH	TA-08	ZSBIO
Akt	10176-2-AP	Proteintech
pAkt	66444-1-Ig	Proteintech
CREB	9197S	Cell signaling
pCREB	9198S	Cell signaling
Synaptophysin	336A-75	Cell marque
BDNF	ab108319	Abcam
TrkB	610,101	BD transduction laboratories
Rac 1	23A8	EMD-millipore corp
pCofilin	3311S	Cell signaling
Cofilin	Sc-376476	Santa cruz
LIMK1	3842S	Cell signaling
ERα	Sc-8005	Santa cruz

### Statistical Analysis

All statistical parameters were calculated using GraphPad Prism software (ver. 7.0; GraphPad Software Inc., San Diego, CA, United States). Values are expressed as means ± standard error of the mean. Results were analyzed by one-way analysis of variance (ANOVA) and repeated measure one-way ANOVA (body weight gain only) followed by Tukey’s post-hoc test. A *p*-value < 0.05 was considered significant.

## Results

### The Effects of Osthole on Body and Uterine Weights in Ovariectomized Mice

Body weight of mice was recorded on days 0, 7, 14 and 21 to evaluate whether OST treatment had an effect on body weight. The pre-surgery body weight between groups showed no significant differences. However, repeated measure one-way ANOVA showed that OVX and OST had a significant effect on body weight gain among the three groups (Figure 1B, F_4.5, 86_ = 7.04, *p* < 0.0001). Mice in the OVX group showed significantly higher body weight gain compared to those in the sham group (Figure 1B, *p* = 0.006), and OST administration reversed this increase in weight gain (Figure 1B, *p* = 0.04) after fourteen days of administration in the OVX + OST group compared with the OVX group on day 21. OVX had a significant effect on uterine weights among the groups (Figure 1C, F _2, 12_ = 6.98, *p* = 0.01). Mice in the OVX group showed significantly lower uterine weight compared to animals in the sham group (Figure 1C, *p* = 0.011), and treatment with OST did not significantly increase uterine weights.

### Osthole Alleviated Anxiety-Like Behaviors in Ovariectomized Animals

**FIGURE 2:**
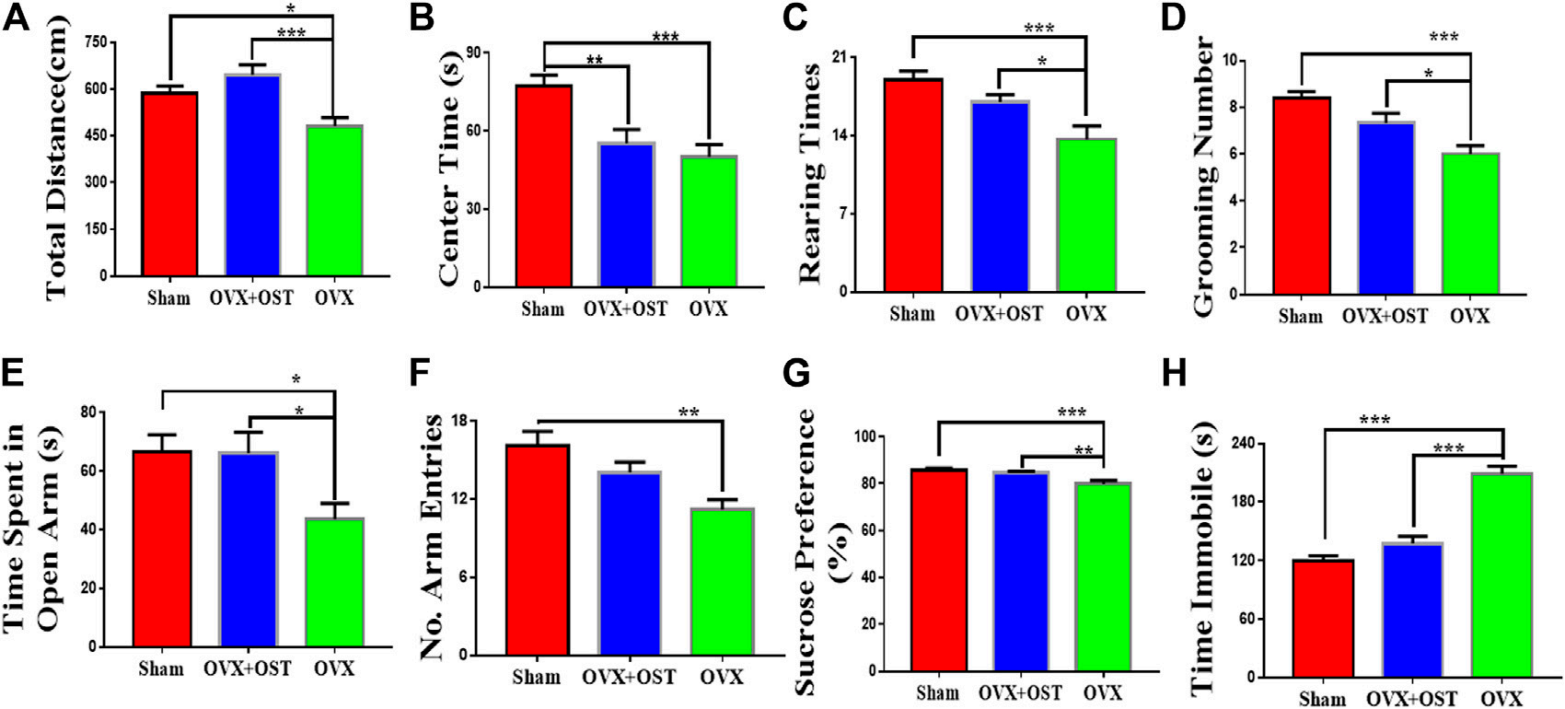
Effects of OVX and OST on anxiety-like and depression-like behaviors in mice **(A–F)** **(A)** The open field test (OFT), OVX mice showed decreased exploratory activity compared to sham controls, and OST reversed this decrease **(B)** OVX significantly decreased the time spent in the center of the open field but OST treatment did not reverse these decreases. **(C,D)** OVX reduced rearing and grooming numbers in the OVX group, and these decreases were reversed by OST treatment. OVX mice spent significant less time in the open arms and made fewer arm entries **(E,F).** Treatment with OST increased the time spent in the open arms but did not increase the number of arm entries in the elevated plus maze test. **(G)** In the sucrose preference test (SPT), OVX caused a reduction in the sucrose consumption but the treatment with OST reversed this effect. **(H)** Mice in the OVX group exhibited an increase in immobility time in the FST while this increase was reversed by OST treatment. Data are represented as mean ± SEM (*n* = 15). *p < 0.05; **p < 0.01; ***p < 0.001.

In the OFT, which is based on natural rodent behaviors including exploration and avoidance of the open area, the total distance traveled, the time spent in the center of the open field (Figures 2A,B), rearing (Figure 2C) and grooming times (Figure 2D) were measured in this test. OVX and OST had a significant effect on the distance traveled (Figure 2A, F_2, 42_ = 6.17, *p* = 0.001) and the time spent in the center of the field among the three groups (Figure 2B, F_2, 42_ = 9.52, *p* = 0.0004). Comparative analysis revealed that the distance traveled (Figure 2A, *p* = 0.03) and the time spent in the center (Figure 2B, *p* = 0.001) in the OVX group were significantly less than in the sham group. OST treatment significantly increased the total distance travelled in the OVX + OST group compared to the OVX group (Figure 2A, *p* = 0.001) but did not reverse the decrease in time spent in the center of the open field (Figure 2B, *p* = 0.72). OVX and OST had a significant effect on the number of rearings (Figure 2C, F_2, 42_ = 8.98, *p* = 0.001) and groomings (Figure 2D, F_2, 42_ = 11.84, *p* < 0.0001). OVX caused a significant decrease in the number of rearings (Figure 2C, *p* = 0.0004) and groomings (Figure 2D, *p* < 0.0001) in the OVX group compared with the sham group. OST treatment reversed the decrease in the number of both rearings and groomings in the OVX + OST group compared with OVX group with vehicle treatment (Figure 2D, *p* = 0.03; Figure 2D, *p* = 0.03).

In the EPM, the time spent by the animals on the open arms was considered to be inversely related to anxiety-like behavior. OVX and OST led to significant differences in the time spent in the open arms (Figure 2E, F_2, 42_ = 4.57, *p* = 0.02) and number of arm entries (Figure 2F, F_2, 42_ = 9.15, *p* = 0.002) among the three groups. Post hoc analyses indicated that the OVX mice spent a significant smaller time in the open arms (Figure 2E, *p* = 0.03) and had fewer entries into the arms (Figure 2F, *p* = 0.001) compared with mice in the sham group. Mice in the OVX + OST group spent more time in the open arms than the OVX group (Figure 2E, *p* = 0.03) but OST treatment did not increase the number of entries into the open arms in OVX + OST group compared with OVX group (Figure 2F, *p* = 0.08).

### Osthole Alleviated Depression-Like Behaviors in Ovariectomized Mice

The FST and SPT were used to assess depression-like behaviors in mice. The SPT is frequently utilized to evaluate the hedonic state in rodents. OVX and OST had a significant effect on sucrose consumption (Figure 2G; F_2, 42_ = 9.18, *p* = 0.001). Further analysis revealed that OVX mice consumed less sucrose than sham mice (Figure 2G, *p* = 0.001). Following treatment with OST, OVX + OST mice showed an increase in sucrose preference (Figure 2G, *p* = 0.01). In the FST, OVX and OST also had a significant effect on immobility time (Figure 2H, F_2, 42_ = 47.5, *p* < 0.0001). OVX mice demonstrated a significant increase in immobility time compared to sham control mice (Figure 2H, *p* < 0.0001). Mice in the OVX + OST group showed a significant decrease in the immobility time in the open field compared to the OVX group (Figure 2H, *p* < 0.0001).

### Osthole Improved Ovariectomy-Induced Memory Impairment in Ovariectomized Mice

**FIGURE 3:**
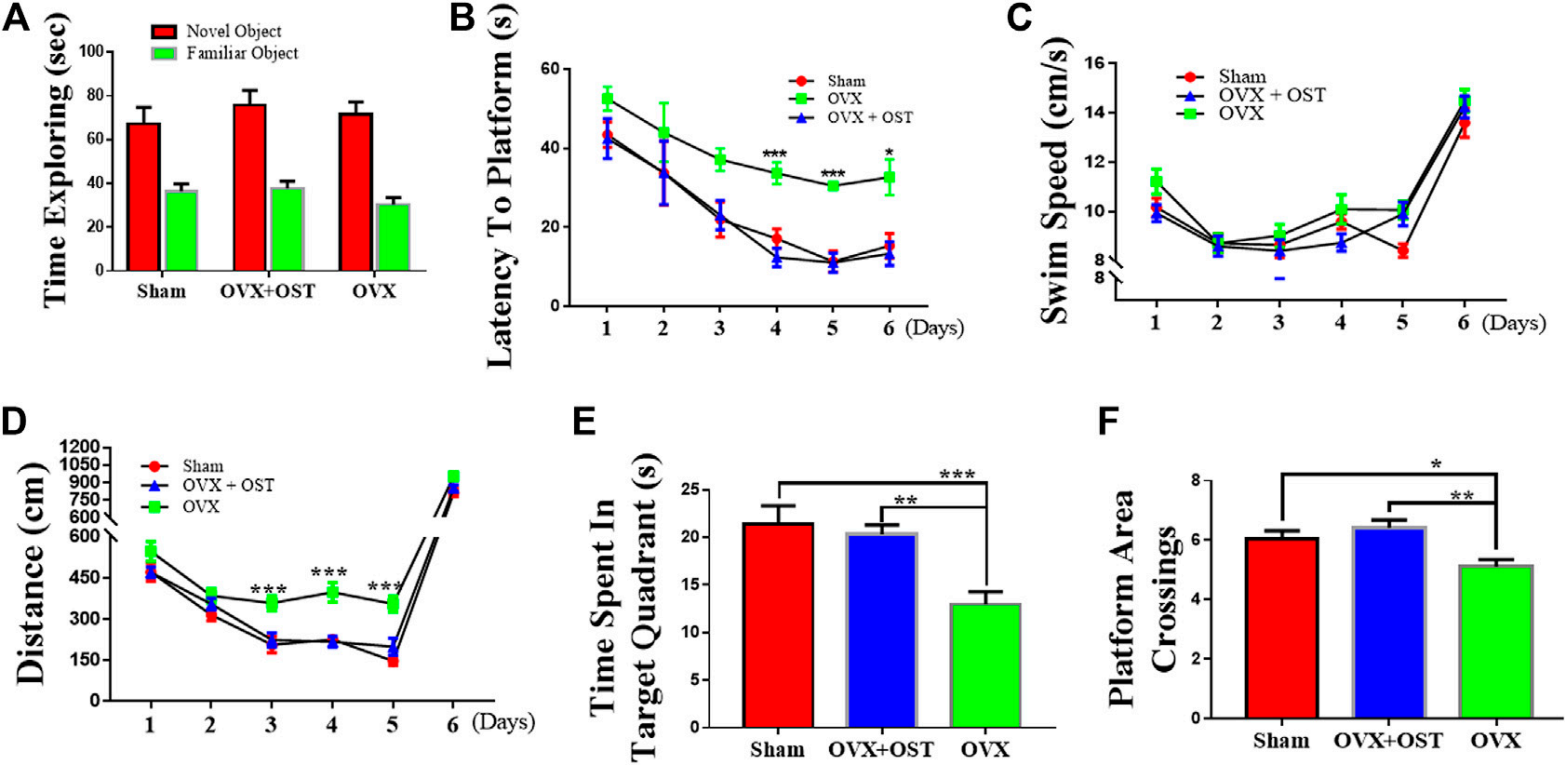
Effects of osthole (OST) on recognition memory **(A)** in the novel object recognition Test (NORT), and spatial learning and memory in Morris Water Maze (MWM) test **(B–F)** in OVX mice. The NORT showed that OVX did not lead to impairment in short-term memory as the time spent in exploring the novel object was not different between the groups **(A)**. **(B–F)** OVX mice showed an increase in latency (spent more time) in finding the platform during the training days (days 3–5), whereas treatment with OST did not alter latency in finding the platform **(B)**. OVX and OST did not have any effect on the swim speed among the three groups **(C)**. OVX mice traveled more distance before finding the platform, while treatment with OST reduced the distance traveled during training on day-3–5 **(D)**. OVX mice made fewer platform crossings **(E)** and spent less time in the target quadrant on the test day 6 **(F)**. The OVX-mediated decrease in the number of platform area crossings and time spent in target quadrant was reversed by OST treatment **(E,F)**. Data are represented as mean ± SEM (*n* = 15). *p < 0.05; **p < 0.01; ***p < 0.001.

To examine the effects of OST on memory function, we performed the NORT and MWM tests, using the NORT to measure recognition memory, and using the MWM test to evaluate spatial learning and memory. OVX and OST did not have a significant effect on the time spent exploring the novel object (Figure 3A, F_2, 42_ = 2.57, *p* = 0.09). Our results showed that OVX and OST treatment did not alter recognition memory (Figure 3A).

In the MWM test, the OVX mice did not differ from the sham group in terms of escape latency to find the platform on days 1–3 during training (directional navigation period). However mice in the OVX group showed an increase in the latency (took a longer time to locate the platform) than animals in the sham control group on days 4–5 during training. Compared with OVX mice, the OST-treated mice had a significantly lower latency to locate the platform on days 4–5 during training (Figure 3B, *p* < 0.05). Neither OVX nor OST treatment had a significant effect on the swim speed among the three groups (Figure 3C, *p* > 0.05), as mice in all three groups swam with a similar speed in their attempt to locate the platform. The total distance traveled before reaching the platform was not significantly different among the three groups on days 1 and 2. Nevertheless, from day 3–5, mice in the OVX group traveled more distance before finding the platform (Figure 3D, *p* < 0.05) than animals in the sham control group, while the OST treatment reversed this increase in the distance traveled. OVX and OST had a significant effects on both the time spent in the target quadrant (Figure 3C; F_2, 42_ = 6.46, *p* = 0.004) and the number of platform crossings on day 6 during the test (Figure 3D, F_2, 42_ = 11.98, *p* < 0.0001). Compared to the sham group, mice in the OVX group showed a decrease in both time spent in the target quadrant (Figure 3C, *p* = 0.046) and the number of platform crossings (Figure 3D, *p* = 0.0001). This decrease was reversed by OST treatment (Figure 3C, *p* = 0.003; Figure 3D, *p* = 0.002). These results showed that OST treatment improved the OVX-induced deficit in spatial learning and memory in OVX + OST mice compared to OVX mice.

### Osthole-Treated Mice Showed Sociability and Preference for Social Novelty in Social Interaction Test

**FIGURE 4:**
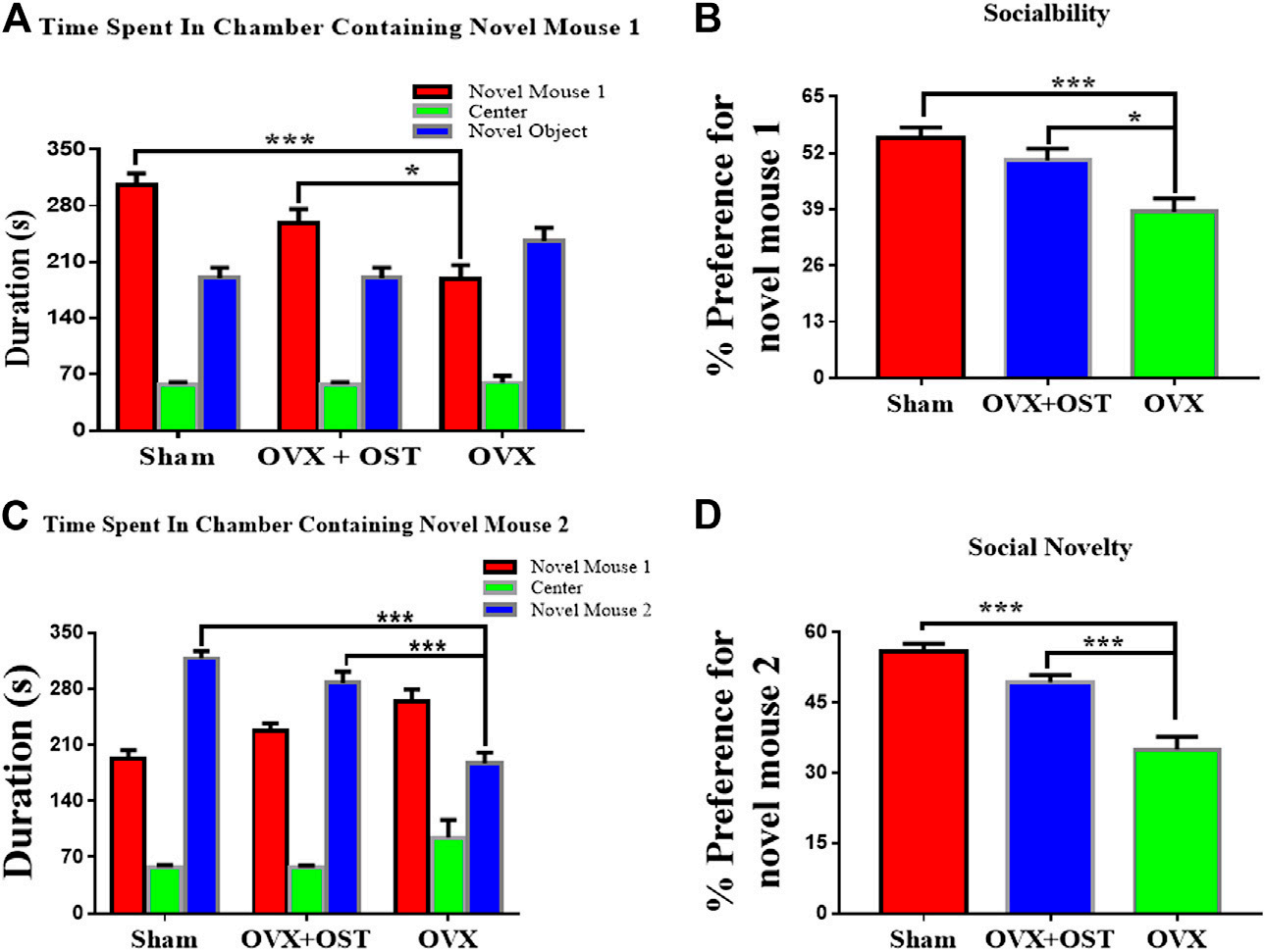
Effects of OST on OVX mice in the three-chamber social interaction test. Mice in the OVX group spent less time interacting with the novel mouse 1 **(A)** and 2 **(C)**, thereby exhibited a decrease in sociability **(B)** and social novelty **(D)**, whereas the mice treated with OST reversed the OVX-mediated decrease. Data are represented as mean ± SEM (*n* = 15). *p < 0.05; **p < 0.01; ***p < 0.001.

We used a three-chamber apparatus to assess sociability (Figures 4A,B). Social interaction test showed that OVX and OST had a significant effect on the time that mice in the three groups interacted with the novel mouse 1 (Figure 4A, F_2, 42_ = 12.96, *p* < 0.0001). Post hoc analysis revealed that the interacting time of OVX mice with the novel mouse 1 was significantly less than that of the sham group (Figure 4A, *p* < 0.0001). OVX mice that received OST injection spent a significant amount of time in interacting with the novel mouse 1 compared to the OVX mice that received vehicle treatment (Figure 4A, *p* = 0.01). In the social novelty test (Figures 4C,D), OVX and OST also had a significant effect on the time that mice in the three groups interacted with novel mouse 2 (Figure 4C, F_2, 42_ = 30.36, *p* < 0.0001). OVX mice spent less time interacting with novel mouse 2 compared to mice in the sham group (Figure 4C *p* < 0.0001). Treatment with OST increased the time spent in interacting with novel mouse 2 (Figure 4C *p* < 0.0001).

### Osthole Reversed Ovariectomy-Mediated Decrease in the Levels of Synaptic Proteins in the Mouse Hippocampus

**FIGURE 5:**
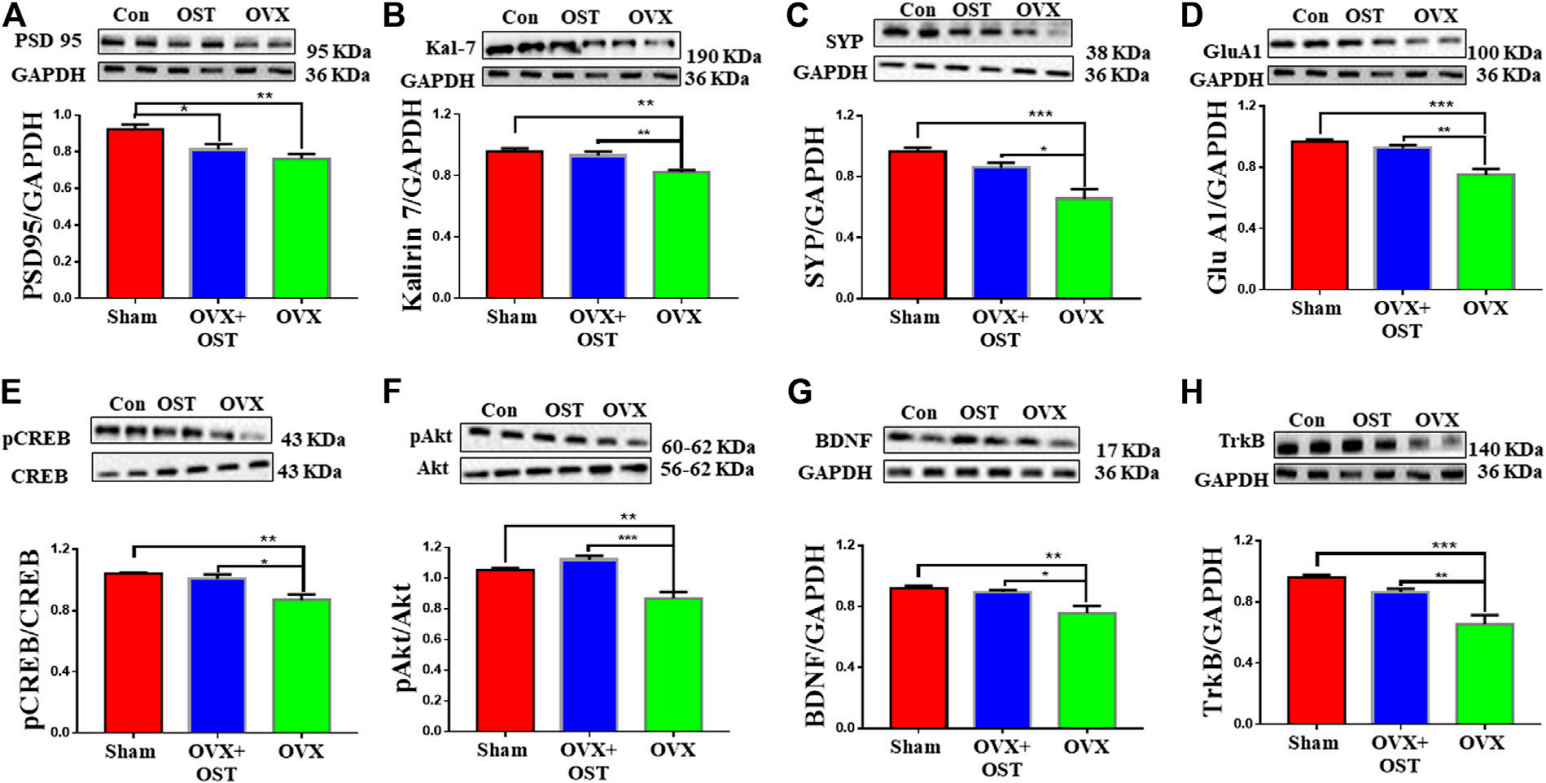
Effects of OST on OVX-induced alterations in the levels of synaptic proteins **(A–D)** and other proteins **(E–H)** in the mouse hippocampus. Expressions of PSD 95 **(A)**, Kalirin 7 (Kal-7) **(B)**, synaptophysin (SYP) **(C)**, GluA1 **(D)**, pCREB/CREB **(E)**, pAkt/Akt **(F)**, BDNF **(G)** and TrkB **(H)** were assessed by Western blot analysis. OVX caused a decrease in the levels of all proteins. Treatment with OST caused a significant increase in the levels of all these proteins with the exception of PSD 95 in the hippocampus of the OVX group compared with the OVX + OST (OST) group. Data are represented as mean ± SEM (*n* = 5). *p < 0.05; **p < 0.01; ***p < 0.001.

To investigate the effects of OST on reduced levels of synaptic proteins induced by OVX, we measured the levels of presynaptic protein synaptophysin (SYP) and postsynaptic proteins PSD-95 and Kal-7 in the mouse hippocampus. OVX and OST had a significant effect on the levels of PSD-95 (Figure 5A, F_2, 12_ = 8.73, *p* = 0.005). Further analysis showed that the PSD-95 level in the OVX group was significantly lower than the sham group (Figure 5A, *p* = 0.004), and the treatment with OST did not significantly increase the level of PSD-95 in the OVX group. OVX and OST significantly affected the levels of Kal-7 (Figure 5B, F_2, 12_ = 11.59, *p* = 0.002). Comparative analysis showed that the Kal-7 level in the OVX group was significantly lower than the sham group (Figure 5B, *p* = 0.002), and treatment with OST reversed this decrease (Figure 5B, *p* = 0.01). There was a significant difference in the levels of SYP protein among the 3 groups (Figure 5C, F_2, 12_ = 12.81, *p* = 0.001). Similar to Kal-7 and PSD-95, OVX also caused a significant decrease in the level of SYP protein in the OVX group compared with the sham group (Figure 5C, *p* = 0.001), and this OVX-mediated decrease in the level of SYP protein was reversed by OST treatment (Figure 5C, *p* = 0.02). We measured the protein levels of AMPA receptor subunit GluA1 in the mouse hippocampus. There was a significant difference in the levels of GluA1 protein among three groups (Figure 5D, F_2, 12_ = 18.89, *p* = 0.0002). Comparative analysis showed that the GluA1 level in the OVX group was significantly lower than the sham group (Figure 5D, *p* = 0.0002) and treatment with OST increased the GluA1 expression to a level similar to the sham control group (Figure 5D, *p* = 0.002).

### Osthole Induced Phosphorylation of Both CAMP Response Element Binding Protein and Protein Kinase B and Reversed Ovariectomy-Mediated Decrease in the Levels of Brain Derived Neurotrophic Factor and Tropomyosin kinase B in the Hippocampus of Ovariectomized Mice

**FIGURE 6:**
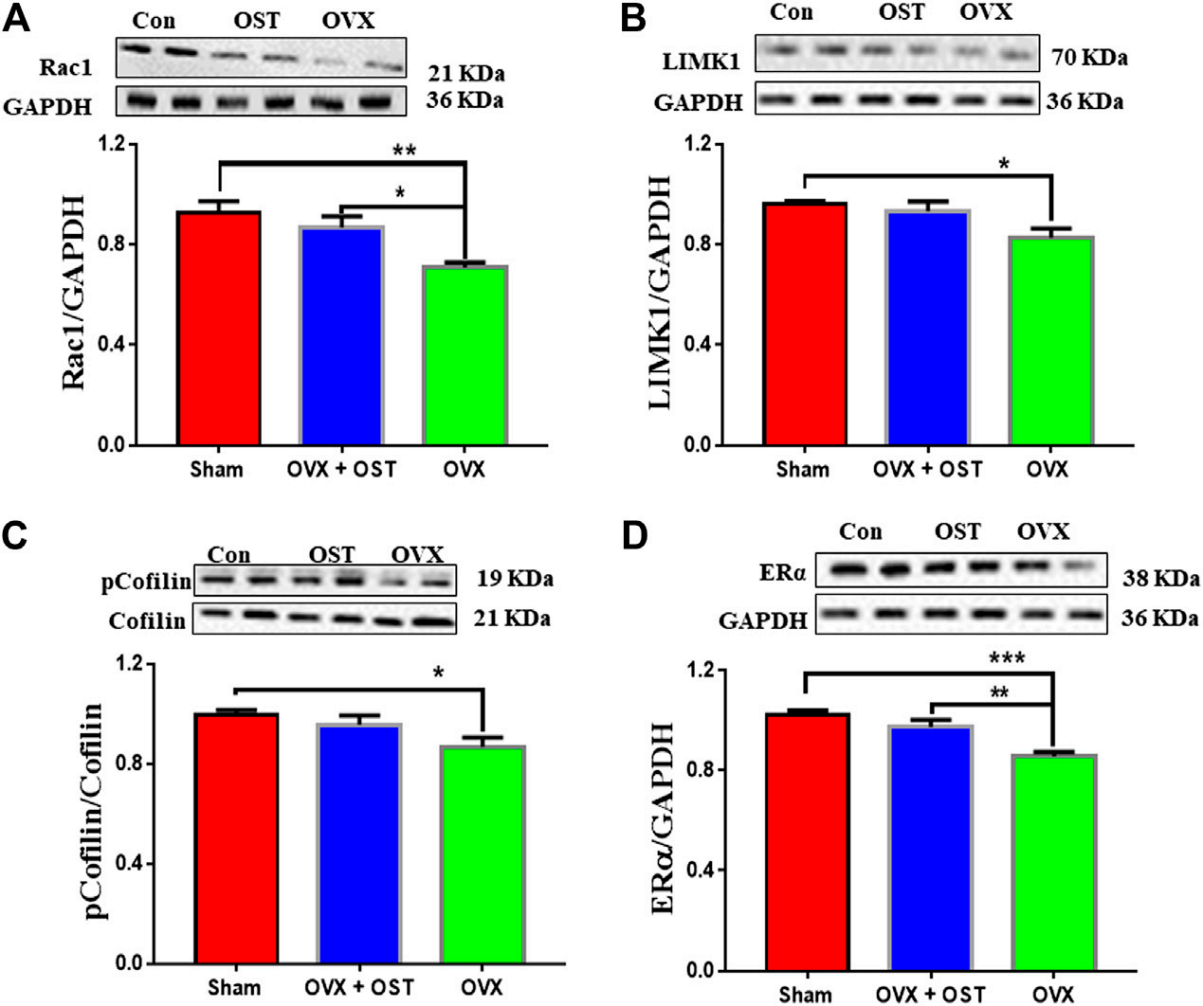
Effects of OST on OVX-mediated alterations in the protein levels of Rac1, LIMK1, pCofilin/Cofilin and ERα in the mouse hippocampus. The levels of Rac1 **(A)**, LIMK1 **(B)**, pCofilin/Cofilin **(C)** and ERα **(D)** were assessed by Western blot analysis. OVX caused a decrease in the levels of these proteins. Treatment with OST reversed the decrease in the levels of Rac1 and ERα, but not LIMK1 and pCofilin in the hippocampus. Data are represented as mean ± SEM (*n* = 5). *p < 0.05; **p < 0.01; ***p < 0.001.

We investigated whether OST activates CREB and Akt in the hippocampus of OVX mice. There was a significant difference in the ratio of p-CREB/CREB in the three groups (Figure 5E, F_2, 12_ = 10.5, *p* = 0.002). Post hoc analysis revealed that the p-CREB/CREB ratio in the OVX group was significantly reduced compared to the sham group (Figure 5E, *p* = 0.003), however, OST significantly restored this reduced ratio (Figure 5E, *p* = 0.01) in the hippocampus in OVX + OST group compared with OVX group. OVX and OST had a significant effect on the ratio of p-Akt/Akt (Figure 5F, F_2, 12_ = 19.22, *p* = 0.0002). Post hoc analysis revealed that the p-Akt/Akt ratio in the OVX group was significantly reduced when compared to the sham group (Figure 5F, *p* = 0.02), and OST significantly restored this ratio (Figure 5F, *p* = 0.0002). The BDNF level in hippocampus was significantly different among the three groups (Figure 5G, F_2, 12_ = 7.68, *p* = 0.007). Post hoc analysis revealed that the BDNF levels in the OVX group was significantly reduced compared to sham control group (Figure 5G, *p* = 0.009), and the reduction was reversed by OST treatment (Figure 6G, *p* = 0.02). The expression of TrkB in the hippocampus was also significantly different among the three groups (Figure 5H, F_2, 12_ = 16.76, *p* = 0.0003). Post hoc analysis showed that OVX resulted in a significant decrease in the TrkB levels in the OVX group compared to sham mice (Figure 5H, *p* = 0.0003) and this decrease was reversed by OST treatment (Figure 5H, *p* = 0.006).

### Osthole Treatment Effects on Levels of Rac1, Cofilin, p-Cofilin, LIM domain kinase 1 and Estrogen Receptor alpha in the Hippocampus of Ovariectomized Mice

For the actin remodeling molecules, one-way ANOVA showed that the levels of Rac1 were significantly different among the 3 groups (Figure 6A, F_2, 12_ = 8.77, *p* = 0.005). OVX produced an obvious decrease in Rac1 (Figure 6A, *p* = 0.004) compared to sham, and OST treatment restored the decrease induced by OVX (Figure 6A, *p* = 0.03). There was a significant difference in the protein levels of LIMK1 (Figure 6B, F_2, 12_ = 4.85, *p* = 0.03). Comparative analysis showed that the LIMK1 level in the OVX group was significantly lower than the sham group (Figure 6B, *p* = 0.03). However, treatment with OST did not reverse the OVX-induced decreases in LIMK1 protein level. OVX and OST had a significant effect on the ratio of p-Cofilin/Cofilin (Figure 6C, F_2, 12_ = 3.89, *p* = 0.049). OVX generated a decrease in the ratio of p-Cofilin/Cofilin (Figure 6C, *p* = 0.045) compared to sham, but the OST changed in this ratio did not reach statistical significance. As shown in Figure 6, OVX and OST significantly affected the ERα level in the hippocampus (Figure 6D, F_2, 12_ = 15.57 *p* = 0.001). Post hoc analysis revealed that the ERα levels in the OVX group were significantly reduced compared to the sham control mice (Figure 6D, *p* = 0.0004), which was reversed by OST treatment (Figure 6D, *p* = 0.006).

## Discussion

OVX (also called surgical menopause) is characterized by reduction in the levels of estrogen, progesterone and testosterone in female rodents. In women, reduced estrogen levels causes depression, sleep disturbance, irritability, anxiety, panic disorders, and cognitive dysfunction (Sherwin, 1998). The current study aimed at evaluating the ameliorative potential of OST against OVX-induced behavioral and molecular changes in mice. The behavioral capacities of mice after OVX were assessed through a wide battery of behavioral tests. This study showed that OVX mice showed various aberrant behaviors including anxiety-behaviors, depression-like behaviors, and cognitive deterioration. Treatment with OST, however, almost completely alleviated these aberrant behaviors. OVX caused widespread alterations in the protein levels of synaptic proteins, ERα, BDNF, TrkB, GluA1, Akt, p-Akt, CREB, p-CREB in the hippocampus. OST treatment consistently reversed OVX-induced alterations in the levels of these proteins in hippocampus.

### Osthole Reversed Ovariectomy-Induced Increase in Body Weight

Body weight gain is strongly linked with bilateral oophorectomy in humans and animals (Roepke, 2009) and it contributes to the extensively reported effect of E2 deficiency (Gonzalez-Garcia et al., 2017). Accordingly, we found that OVX mice gained more weight compared to sham control animals over the experimental period. One of the risk factors for mood disorders and cognitive impairment is excessive body weight (Zanini et al., 2017). OVX-induced body weight gain is prevented with E2 replacement (Roesch, 2006). OST treatment was able to reverse the OVX-induced body weight gain. Previous reports showed that the activation of ERs is effective in preventing body weight gain in OVX mice (Pedersen et al., 2004; Yepuru et al., 2010). OST treatment-attenuation of body weight gain in OVX mice is consistent with results of the previous studies (Yepuru et al., 2010), as OST also significantly increased the expression levels of ERα in the hippocampus of OVX mice.

### Osthole Improved Ovariectomy-Induced Deficits in Spatial Learning and Memory, Recognition Memory and Sociability

The MWM test, a hippocampus-dependent memory task, is commonly used to evaluate cognitive status in rodents. The directional navigation training trials are utilized to evaluate spatial learning, and the probe trials when the platform is absent are used to examine whether the animal remembers the location of the platform, which indicates memory capability. The increased latency in the MWM showed OVX-induced impairment in spatial learning in OVX mice, in agreement with a previous report (Gresack and Frick, 2006). Time spent in the target quadrant and the number of platform crossings also provide more robust measures of memory. Spatial memory is determined by preference for the platform area when the platform is absent during the test. OVX mice spent less time in the target quadrant and made less platform crossing compared to the sham control mice, a deficit reduced by OST treatment. . These results showed that OST treatment improved the OVX-induced deficit in spatial learning and memory. Our results are similar to clinical findings where cognitive deficits induced by estrogen deficiency can be improved by estrogen therapy (Bagger et al., 2005). However, a possible advantage of using OST vs estrogen for treatment of menopausal symptoms is that OST may not harbor the same side effects as traditional hormone therapies (Beral et al., 2015).

The present study showed that the ability to recognize other individuals is hampered in OVX mice just as previously reported for mice in the three-chamber social interaction test (Karlsson et al., 2015). Thus, the lack of estrogen in the mice led to deficits in recognition memory and sociability. This deficit in sociability was reversed by treatment with OST. Even though OVX may induce cognitive dysfunction in rodents, OVX females in our experiment exhibited no memory impairment in the object recognition task (non-spatial recognition memory), in accordance with a previous report (Liu et al., 2019), and the OST-treated group did not outperform OVX mice. A large number of studies have shown that estrogen deprivation leads to memory impairment in rodents (Phan et al., 2015; Hwang et al., 2017). However, OVX females in our experiment did not exhibit recognition memory impairment after 4 weeks of OVX (it was 4 weeks after OVX when NORT was performed), as the sham and OST-treated groups did not outperform these OVX mice in the time spent exploring the novel object. Our results is in agreement with a previous report that 4 week OVX did not alter recognition memory (Tao et al., 2020) but not in agreement with another study in which 4 week OVX impaired recognition memory in the NORT in mice. Actually, there is a variation in the duration of OVX across studies reporting conflicting results (Fonseca et al., 2013; Mitra et al., 2016; Aggarwal et al., 2019; Tao et al., 2020), and this seems to be a contributing factor for these conflicting findings. For example, impaired recognition memory in mice in the NORT was found after 1 week OVX (Mitra et al., 2016). However, another conflicting study did not find a significant difference in mouse recognition memory between the sham and OVX groups after 1 week OVX, but a significant difference was observed after 6 weeks OVX (Fonseca et al., 2013). However, a lack of impaired recognition memory in OVX mice in our study may result from the short interval (3 h) between training and testing.

### Osthole Alleviated Ovariectomy-Induced Anxiety- and Depression-like Behaviors

The incidence of anxiety in women has long been linked to changes in the levels of circulating estrogen across the reproductive lifespan. Anxiety and depression symptoms occur when the levels of estrogen drop after surgical menopause (oophorectomy) (Rocca et al., 2008) and in postmenopausal women (Sahingoz et al., 2011). OVX is a strong risk factor for anxiety and depression. In the present study, we used the EPM and OFT to evaluate the effects of OST on anxiety-like behavior in mice after OVX. OVX caused a decrease in time spent in the open arms. Administration of OST alleviated these anxiety-like behaviors seen in OVX mice, showing an anxiolytic-like effect of OST. In the OFT, OVX led to decreases in both distance traveled and the amount of rearing and grooming in OVX mice compared to sham control. These decreases were reversed by the administration of OST. The reduced distance traveled reflects a decrease in locomotor activity in OVX mice. (Bailey and Crawley, 2009). Rearing and grooming behaviors are the indexes of the emotional state of mice (Gilad et al., 2000). OVX-mediated decrease in the number of rearing and grooming accompanied anxiety-like behaviors in the EPM, which suggests that this decrease indicated a high level of anxiety-like behavior in mice (Bouwknecht et al., 2007), in agreement with previous reports in similar studies (Rodríguez-Landa et al., 2017; Puga-Olguín et al., 2019). It is worth noting that rearing and grooming may not be the best measure for anxiety-related behavior as conflicting reports show that rearing behavior is either anxiolytic or anxiogenic (Seibenhener and Wooten, 2015; Kraeuter et al., 2019); decreased rearing (Borta and Schwarting, 2005) and grooming (Lund et al., 2005; Zhang et al., 2019) are less anxious behaviors, and increased rearing (Borta and Schwarting, 2005) and grooming (Lund et al., 2005; Zhang et al., 2019) are indicators of heightened anxiety-like behavior. The OVX-mediated alteration in grooming and rearing behavior was prevented by OST treatment.

Since ovarian hormones exert antidepressant and anxiolytic actions (Recamier-Carballo et al., 2012) and a prolonged deprivation of ovarian secretions increases risk for the development of depression and anxiety (Rocca et al., 2008) we further investigated the effects of OST on depression-like behaviors in OVX mice using the FST. This test evaluates hopelessness or despair, a major symptom in patients with depression. This type of symptom is characterized by an increase in immobility times in the FST in rodents, and it is used as an index of depression-like behaviors. In the current study, OVX mice exhibited an increase in immobility time in the FST compared with sham control mice. Repeated administration of OST reversed the OVX-induced increase in immobility time in the FST. Our results are in agreement with an earlier study showing that OVX induces an increase in immobility time in the FST in mice (Heydarpour et al., 2013). The SPT is an assay of anhedonia and is sensitive to treatment with antidepressants. In this task, the absence of a preference for more palatable sweetened water over normal drinking water in rodents reflects a defective reward system and thus an anhedonic state. Our results showed that OVX caused a decrease in sucrose preference in mice, in the line with a previous report (Zhou et al., 2019), and chronic OST treatment reversed the OVX-induced decrease in sucrose consumption. Notably, OST treatment increased sucrose preference in OVX mice almost to the level of sham control mice. These results show that the absence of ovarian hormones is a risk factor for the development of depression- and anxiety-like behaviors, in agreement with previous preclinical (Zhou et al., 2019) and clinical studies (Rocca et al., 2008). This effect of OST is similar to E2. E2 replacement also alleviates OVX-induced depression-like behaviors in OVX mice compared with sham control mice (Bekku et al., 2006). To the best of our knowledge, this is the first study showing alleviation of anxiety- and depression-like behaviors in OVX mice by OST treatment.

### The Mechanisms Through Which Osthole Rescued Ovariectomy-Induced Cognitive Deficit and Alleviated Anxiety- and Depression-like Behaviors

Our results showed that OVX caused a decrease in the levels of ERα in the hippocampus, in agreement with a previous report (Wang et al., 2016). Interestingly, the OVX-mediated decrease in ERα level was reversed by the OST treatment. The hippocampus is an area in the brain that plays a crucial role in cognitive functions (McEwen, 2002), and both ERα and ERβ are highly expressed in the hippocampus (Register et al., 1998). ERα is the predominant receptor in the hippocampus responsible for synaptogenesis (Foster, 2012). Similar to estrogen, the ERα agonist, PPT, caused an improvement in the OVX-mediated cognitive deficit (Phan et al., 2011). The level of endogenous E2 in the hippocampus is much higher than circulating E2 (Kato et al., 2013), and a significant level of E2 is found in hippocampus even after OVX (Kato et al., 2013). Interestingly, endogenous E2 plays an essential role in hippocampal synapse formation (Kretz et al., 2004). Dendritic spine/synapse number is associated with cognitive function. Therefore, endogenous hippocampal estrogen may play a role in the OST-mediated rescue in cognitive deficit induced by OVX. Therefore, OST-mediated increase in the ERα level may contribute to improved cognitive function in OVX mice. Our results are the first showing that intraperitoneal administration of OST improved spatial learning and memory in OVX mice, and that the effect of OST is comparable with the effect of E2.

### Role of Osthole-Mediated Reversal of the Ovariectomy-Induced Decrease in the Levels of Synaptic Proteins

**FIGURE 7:**
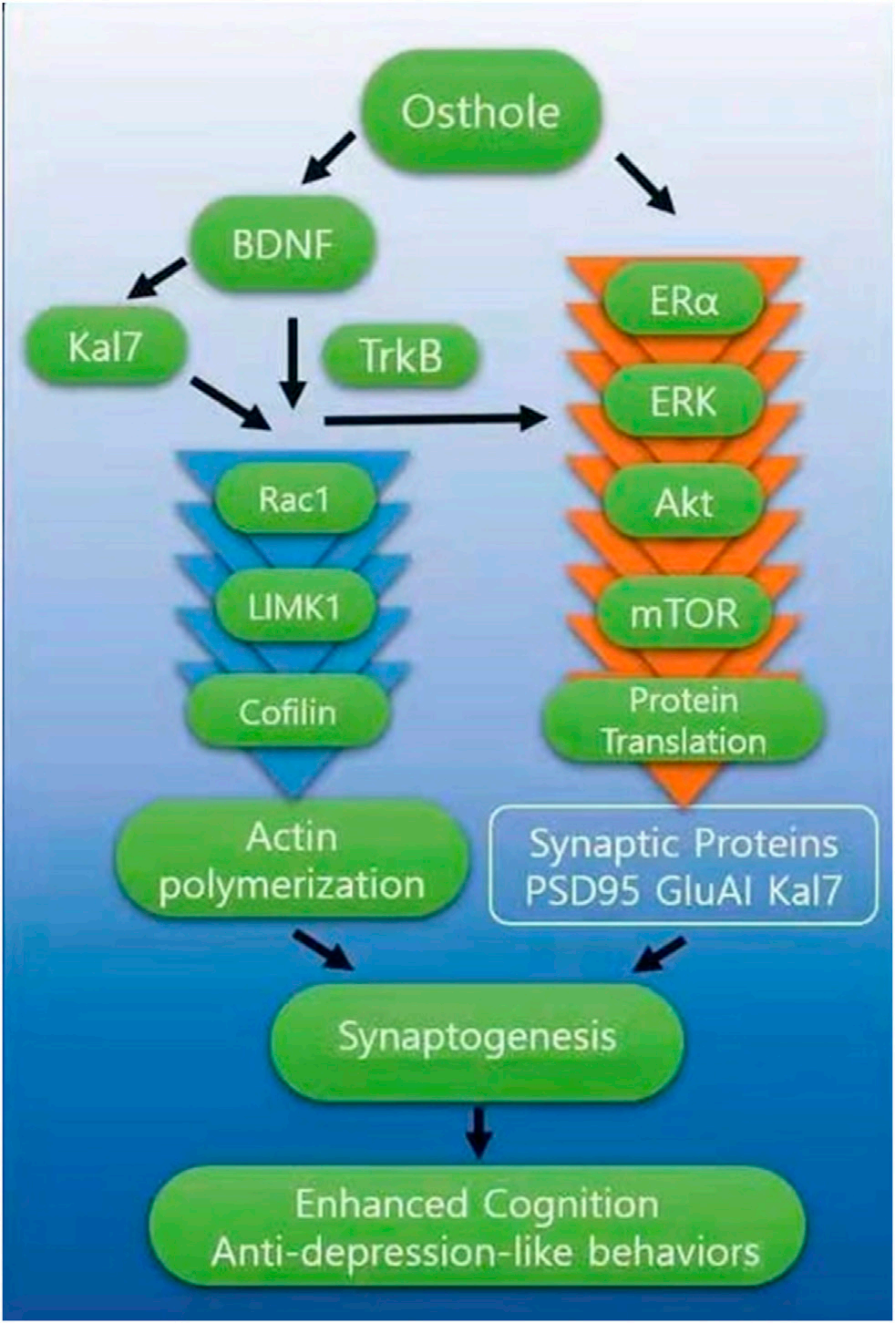
The figure above summarizes the effects of OST in OVX mice. Chronic OST treatment led to changes in the levels of synaptic proteins in the hippocampus and at least some of these effects require BDNF/TrkB signaling. OST interacts with BDNF and ERα, leading to activation of downstream signals to improve cognitive impairment and ameliorate depression-like behaviors observed in OXV mice. OST caused increase in the release of BDNF, which then binds to and activates its surface receptor, TrkB then activates one of its two major downstream signaling cascades, ERK-Akt. This pathway converges onto the mammalian/mechanistic target of rapamycin (mTOR), a key regulator of protein synthesis and synaptic plasticity. This event leads to disinhibition of synaptic protein translation (e.g., GluA1, PSD95, Kal7), which leads to synaptogenesis. The interaction of BDNF with Kal7 also influenced the Rac1/LIMK1/Cofilin pathway. OST-mediated changes in the levels of Rac1 also suggest that OVX-induced perturbation of hippocampal synaptic connectivity was reversed by treatment with OST, through functional modifications of neuronal networks in the brain. This suggests that the effects of OST on hippocampus-related behavior is in part due to the effects on the connectivity of neurons in this brain region.

Our results showed that OST caused an increase in the levels of synaptic proteins including, Kal-7, SYP and GluA1 plus increased levels of BDNF, TrkB, p-CREB and p-Akt in the hippocampus of OVX mice. The phosphorylation of CREB stimulates the synthesis of proteins such as SYP and PSD-95, which are related to synapse formation and memory enhancement (Mizuno et al., 2002). SYP is a presynaptic protein and is closely connected with cognitive function (Stockert et al., 2012). OST-induced increase in SYP protein level in the hippocampus may contribute to the amelioration of memory impairment in OVX mice. The OST-induced increase in the levels of synaptic proteins including, GluA1, Kal-7 in hippocampus may lead to accelerated synapse formation thereby enhancing cognitive function (Figure 7). Another possible mechanism through which OST caused an increase in these synaptic proteins is via its interaction with BDNF/TrkB; increased BDNF induced by OST could be released into synapses, where BDNF binds to and activates its TrkB receptor that then activates one of its two major downstream signaling cascades, the phosphatidyl-inositol-3-kinase (PI3K-Akt) pathway. This pathway converges onto the mammalian/mechanistic target of rapamycin (mTOR), a key regulator of protein synthesis and synaptic plasticity. This event leads to disinhibition of synaptic protein translation (e.g., GluA1, PSD95), which leads to synaptogenesis and then enhancement in learning and memory. Interaction of Kal-7 with PSD95, GluA1 and TrkB (Mandela and Ma, 2012; Yan et al., 2016) plays an essential role in the formation of excitatory synapse and dendritic spines (Ma et al., 2008; Ma et al., 2011). Kal-7 action is downstream of BDNF (Yan et al., 2016), since BDNF cannot stimulate neurite outgrowth in cultured cortical neurons when Kal-7 is deleted in these neurons (Yan et al., 2016). In addition, BDNF loses its ability to enhance spine formation when the endogenous Kal-7 level is decreased in hippocampal neurons (Li XM and MA XM unpublished). Alterations in dendritic spines and synapse are closely associated with learning and memory, anxiety- and depression-like behaviors (Qiao et al., 2016; Zagrebelsky et al., 2020). Since spine density and morphology were not studied in this work, we are unable to draw a firm conclusion on the relationship between increased levels of synaptic protein and spine density/morphology, but it is likely that the OST-mediated increase in the levels of these proteins contributes to the OST-mediated improvement in spatial learning and memory, anxiety- and depression-like behaviors in OVX mice. Thus, OST has a great potential as a therapeutic drug that works in the brain to rescue cognitive deficits.

### Role of Osthole-Mediated Reversal of the Ovariectomy-Induced Decrease in the Levels of Rac1 LIMK1 and Cofilin

Our results showed that OVX resulted in decreased levels of phosphorylated (p)-CREB, p-AKt, Rac1, LIMK1 and p-cofilin in the hippocampus of OVX mice, and these decreases were reversed by OST except for LIMK1 and p-cofilin. The Rac1/LIMK1/Cofilin pathway may play an important role in enhancing cognitive function by OST. Rac1, a small GTPase, plays a key role in regulating the actin cytoskeleton; Rac1 activation increases spine density and enhances learning and memory (Nakayama et al., 2000). Rac1 is activated by Kal-7 (Schiller et al., 2005), and the OST-meditated increase in the Kal-7 level may play an important role in Rac1 activation. Phosphorylation of LIMK1, a downstream target of Rac1 controls the polymerization of actin through the actin binding protein. The regulation of these important proteins by OST may play a key role in reversing the OVX-induced alterations in behaviors by OST. Phosphorylation-dependent activation of several signal transduction pathways is associated with cognition (Monti et al., 2005). These pathways include the LIMK pathway, the cAMP response element binding protein (CREB) pathway, and the phosphatidyl-inositol-3-kinase (PI3K)/Akt pathway. Akt in particular is a key signal transduction intermediate that is relevant to learning and memory. Thus, these findings suggest that activation of the Akt pathway is well positioned to facilitate the OST-stimulated enhancement in learning and memory, as reported in the current study. In addition, interaction of OST with BDNF may also contribute to OST’s ability to rescue the OVX-induced cognition deficit and alleviate depression-like behaviors since BDNF also plays a key role in cognition (Cunha et al., 2010) and in alleviating depression-like behaviors (Qiao et al., 2017). A decrease in BDNF expression is closely associated with menopause-related anxiety, depression, and cognitive deterioration (Van Kempen et al., 2014). BDNF plays an essential role in maintaining learning and memory via its TrkB receptor (Cunha et al., 2010), exerting its effects by activating the downstream signaling pathways including PI3K/Akt and mitogen-activated protein kinase (MAPK) (Bonni et al., 1999; Brunet et al., 2001). These findings revealed that OST-mediated improvement in cognition deficit and amelioration in anxiety- and depression-like behaviors induced by OVX, are all closely associated with the Rac1/LIMK1/Cofilin, p-CREB and BDNF/TrkB/Akt pathways.

## Conclusion

Results from the present study showed that chronic OST treatment resulted in an amelioration of cognition deficits and anxiety- and depression-like behaviors observed in OVX mice. The OST-mediated reversal of OVX-mediated decrease in the levels of Kal-7, SYP, GluA1, BDNF, TrkB, Rac1, p-CREB and p-AKt may contribute to OST-mediated behavioral improvements. Our findings showed that OST is a promising candidate for use as an alternative choice in hormone replacement therapy.

## Data Availability

The original contributions presented in the study are included in the article/Supplementary Material, further inquiries can be directed to the corresponding authors.

## Abbreviations

OST, osthole; OVX, ovariectomy; ERα, estrogen receptor alpha; BDNF, brain derived neurotrophic factor; TrkB, tropomyosin kinase B; CREB, cAMP response element binding protein; Akt, protein kinase B; Rac1, Ras-related C3 botulinum toxin substrate 1; LIMK1, LIM domain kinase 1; PSD-95, post synaptic density protein 95; APP, Amyloid-beta precursor protein; NORT, novel object recognition test; MWM, Morris water maze; SPT, sucrose preference test; OFT, open field test; EPM, elevated plus maze; FST, forced swim test; GAPDH, Glyceraldehyde 3-phosphate dehydrogenase; PI3K, phosphatidyl-inositol-3-kinase.
